# Malignant Transformation in a Mature Cystic Teratoma of the Ovary: An Unusual Case Presentation

**DOI:** 10.7759/cureus.37304

**Published:** 2023-04-08

**Authors:** Shubha Dadhich, Shubha S Manjunath, Ramji Swaminathan

**Affiliations:** 1 Obstetrics and Gynecology, NMC Specialty Hospital, Abu Dhabi, ARE; 2 Anesthesiology, NMC Specialty Hospital, Abu Dhabi, ARE

**Keywords:** laparotomy, borderline ovarian tumours, ovarian torsion, benign mature cystic teratoma, mucinous cystadenocarcinoma

## Abstract

Mature cystic teratoma is the most common type of ovarian germ cell tumor. Malignant transformation is a rare complication in 1-3% of cases, especially in post-menopausal women. The most common type of malignant transformation by histology is squamous cell carcinoma, followed by adenocarcinoma, carcinoid tumor, melanoma, and sarcoma. Diagnostic difficulties exist due to non-specific findings. No clinical, radiological, or biological signs are specific to malignant transformation. Most patients are diagnosed in advanced stages and have poorer outcomes. Staging of the disease is an important prognostic factor, with early diagnosis and treatment being critical for improved survival. Here, we report a rare case of mature cystic teratoma presented as torsion, postoperatively diagnosed as mature cystic teratoma with borderline mucinous cystadenoma and focal intra-epithelial malignant transformation.

## Introduction

According to the WHO classification of ovarian tumors, a mature cystic teratoma is the most common germ-cell tumor. Malignant transformation occurs in about 1-3% of mature cystic teratoma patients, especially post-menopausal women. Borderline ovarian tumors are neoplastic growths of epithelial origin and are characterized by up-regulated cellular proliferation and minimal nuclear atypia but no destructive stromal invasion [1]. Taylor first described these tumors in 1929 as “semi-malignant” ovarian tumors involving the peritoneum, with a surprisingly good prognosis. It was subsequently recognized by the International Federation of Gynecology and Obstetrics (FIGO) in 1971 as tumors of “low malignant potential” and considered distinct from ovarian carcinomas [2]. The 2014 WHO Classification of Tumors of the Female Genital Organs uses “borderline tumor” alternatively with “atypical proliferative tumor.” These borderline mucinous tumors or atypical proliferative mucinous tumors of the ovary constitute nearly 15% of all mucinous epithelial tumors [3]. These tumors are the second most common type, forming approximately 30-50% of borderline ovarian tumors. Most mucinous borderline tumors have an excellent prognosis, with overall survival approaching 95-100% [4].

## Case presentation

**Figure 1:**
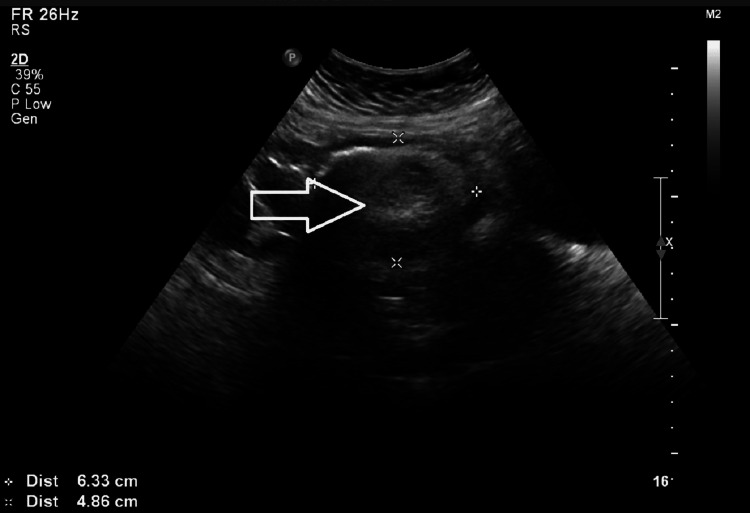
Ultrasound

A 49-year-old perimenopausal woman with two previous vaginal deliveries came to the emergency room with acute pain in the left lower abdomen radiating from the left flank for a day. An urgent workup was done, and medical management was done. She had a history of recurrent episodes of left lower abdomen pain. Her menstrual cycle was irregular, with a history of secondary amenorrhoea for four months; she was a known case of essential hypertension and type 2 diabetes mellitus on irregular medication. A complete abdomen scan revealed a *well-defined hyperechoic lesion in the pelvis measuring approximately 64 x 50 mm, the possibility of ovarian dermoid" (Figure 1).

**Figure 2:**
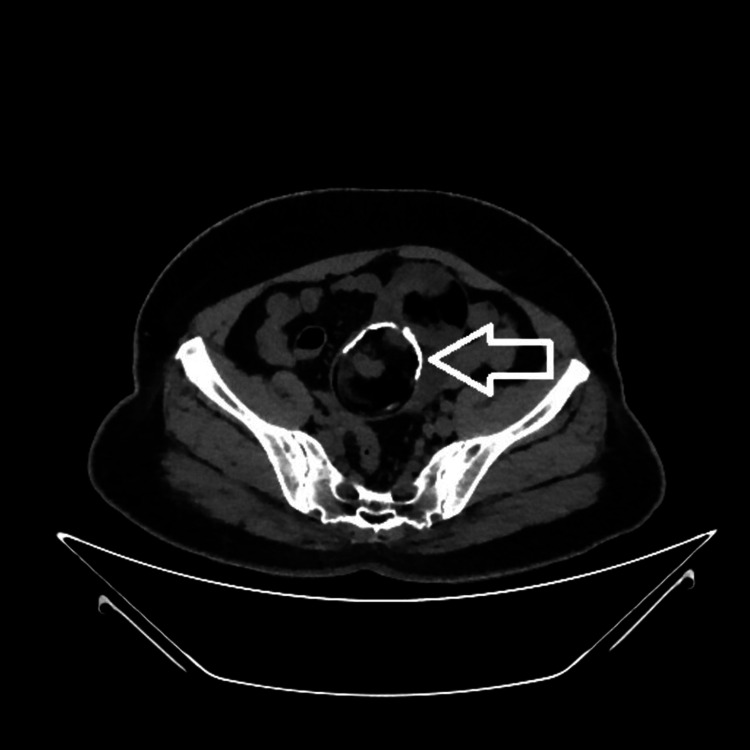
Plain axial view

**Figure 3:**
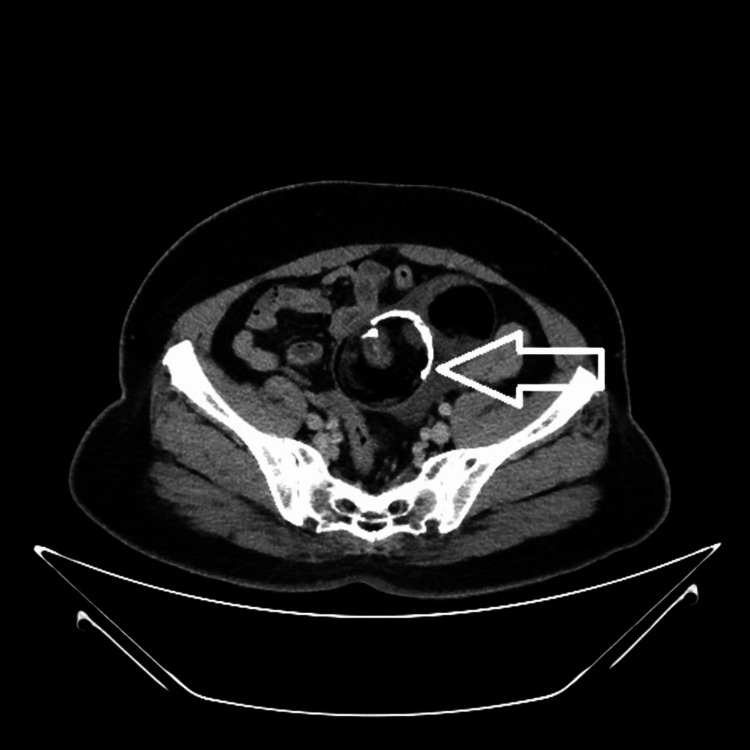
Contrast axial view

**Figure 4:**
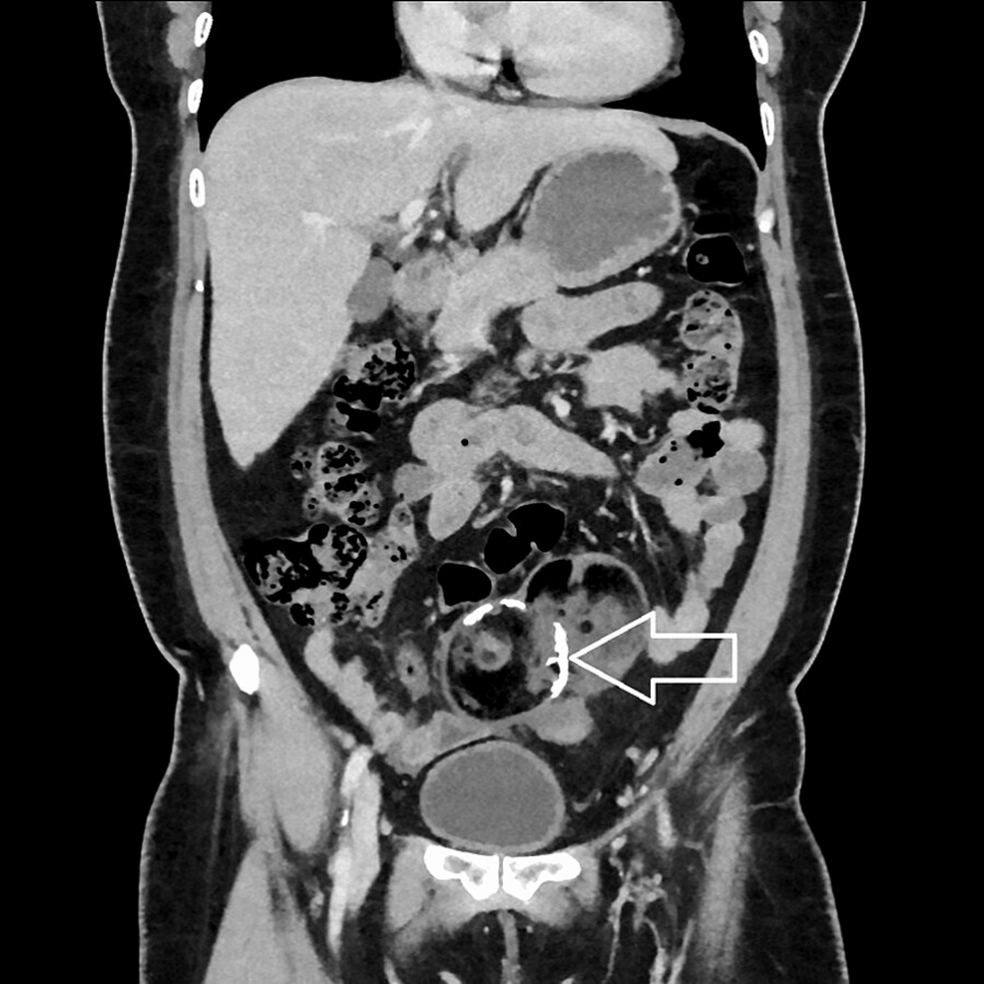
Coronal contrast view

**Figure 5:**
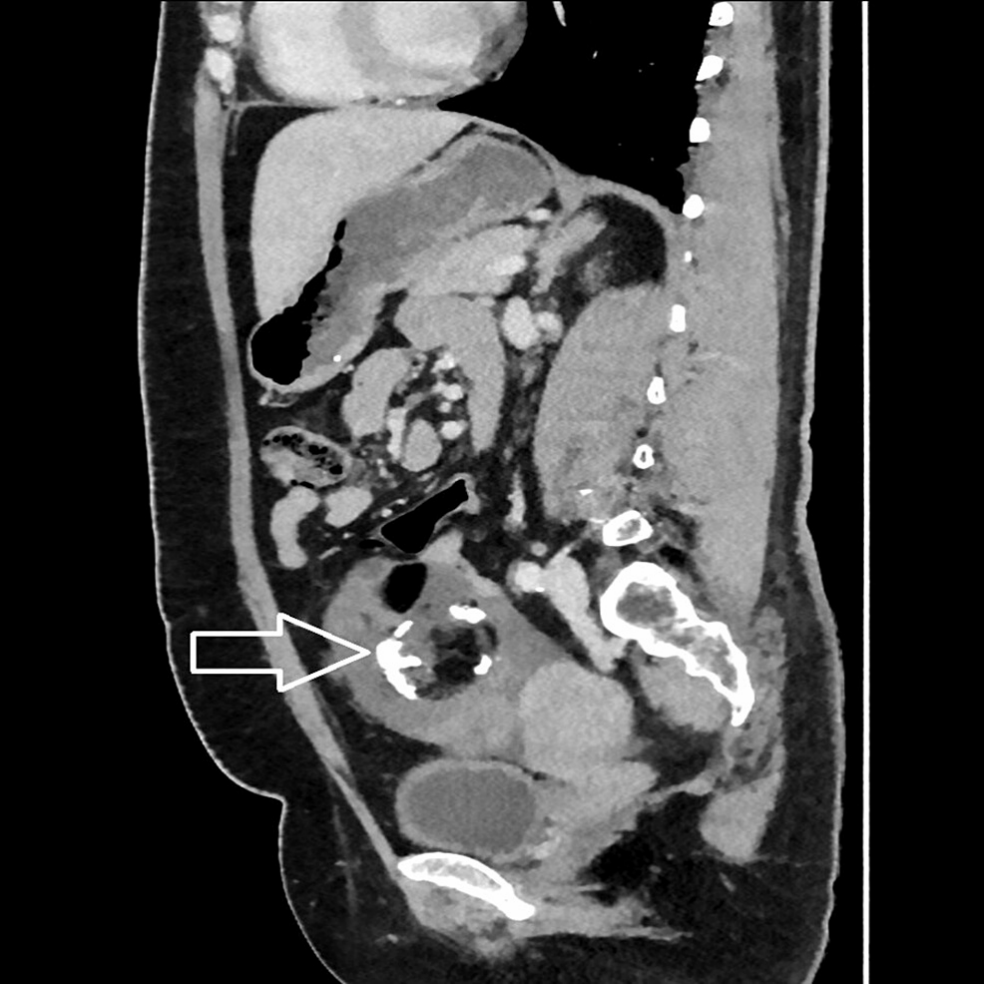
Contrast sagittal view

A computed tomography (CT) scan revealed a well-defined, lobulated, predominantly fat-density lesion measuring approximately 112 x 62 x 108 mm in the lower abdomen superior to the uterus. It revealed fluid density locules, soft tissue components, calcific sand densities, and peripheral density wall calcification, showing enhancing septa and solid components. The lesion was adherent to adjacent small bowels with minimal fluid in the pelvis. Ovaries could not be made out separately. Radiologically, a diagnosis of mature cystic teratoma was made (Figures 2-5).

Ovarian tumor markers were advised and showed normal values (Table 1).

**Table 1:** Ovarian tumor markers

Biochemical marker	Patient value	Normal range
CA 125	16 U/ml	0-35 U/ml
Alpha-Fetoprotein	0.80 ng/ml	< 8.1 ng/ml
Carcino Embryonic Antigen	2.07 ng/ml	upto 2.5 ng/ml
Beta Human Chorionic Gonadotrophin (Beta HCG)	0.29 U/L	< 5.0 U/L
Lactate Dehydrogenase (LDH)	169 U/L	< 250 U/L

After a detailed discussion of reports with the patient and family, informed consent was taken for left salpingo-oophorectomy/total abdominal hysterectomy with left salpingo-oophorectomy with the removal of ovarian mature cystic teratoma in view of large mature cystic teratoma with recurrent episodes of pain. Pre-operative laboratories were normal. 

**Figure 6:**
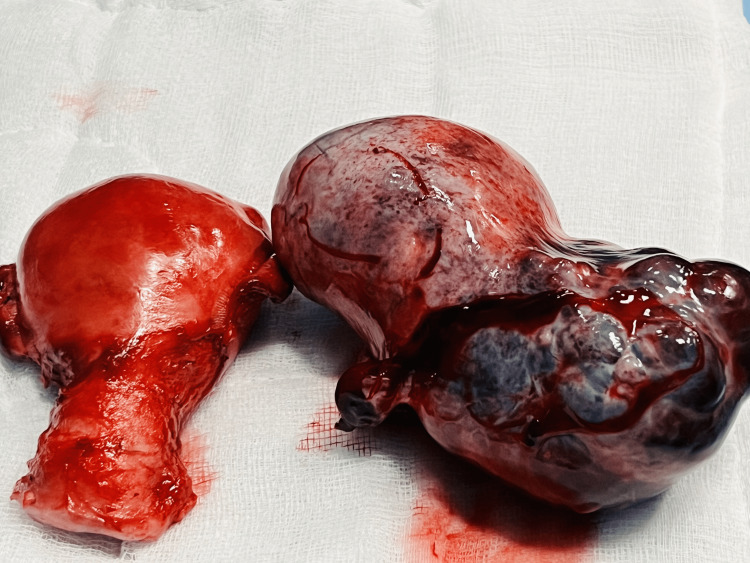
Surgical specimen

Her intraoperative findings showed that the uterus was bulky, globular, and adenomyotic in appearance. A large adnexal mass, twisted twice on its pedicle (gangrenous mass of 10x11 cm), was noted on the left side of the uterus. The intraoperative diagnosis was twisted mature cystic teratoma of the left ovary (Figure 6). Her right side tubes and ovaries were normal. We proceeded with a total abdominal hysterectomy and left salpingo-oophorectomy under general anesthesia. The postoperative period was uneventful. She was discharged home on postoperative day 3 in satisfactory condition.

**Figure 7:**
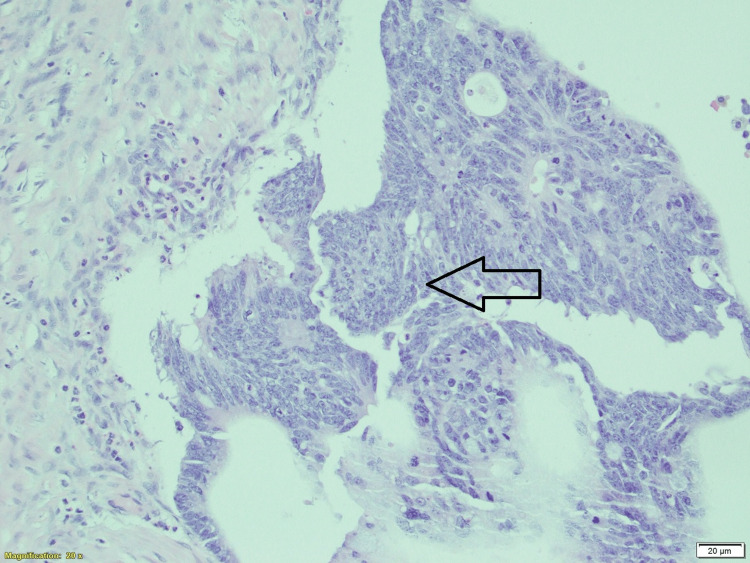
Microphotograph shows an intra-mucosal carcinoma arising in a mucinous borderline tumor in teratoma

Her histopathology report showed mature cystic teratoma with a borderline mucinous tumor and focal intraepithelial carcinoma. A left ovarian tumor of 12 x 6.5 x 5.5 cm was identified. The uterus, left ovarian surface capsule, and left fallopian tube serosa were intact. The histological grade was a borderline tumor. The pathological stage was identified as pT1a and FIGO Stage IA. Immunohistochemistry revealed CK7 (Cyto Keratin 7) - mucinous tumor negative, ovarian surface positive, CK20 - mucinous tumor epithelium positive (Figure 7). CK20 is expressed more in primary ovarian tumors while CK7 is more expressed in metastatic ovarian tumors. In our case, immunohistochemically, CK20 expression was mucinous tumor epithelium positive and CK7 expression was mucinous tumor negative. This concludes its primary ovarian origin, not metastatic. This patient was diagnosed with FIGO stage 1A, with an excellent prognosis and a five-year survival rate of 95-97%. The patient was referred to a gyne oncologist for a second opinion. Expectant management with follow-up after three months was advised.

## Discussion

The first description of a patient with a borderline mucinous tumor associated with a mature cystic teratoma was reported in 1988. A mature cystic teratoma's borderline tumorous change has rarely been reported [5]. Borderline ovarian tumors are distinct groups of neoplasms that demonstrate higher proliferative activity than benign neoplasms, which do not show stromal invasion [6]. Borderline ovarian tumors are more likely to be seen in younger women than older women [6].

Mature cystic teratomas are one of the most common tumors seen in females of reproductive age. A malignant change in benign cystic teratomas is seen in 1-3 % of cases [3,7]. The rate of malignant transformation in mature cystic teratomas increases with age and is much higher in the postmenopausal age group. Our patient is a 49-year-old perimenopausal lady diagnosed with a borderline mucinous tumor and focal intraepithelial carcinoma arising in a mature cystic teratoma who presented with ovarian torsion. Torsion of the fallopian tube or ovary presents as a gynecological emergency in 2-7% [8]. Our patient presented with acute left lower abdominal pain to the emergency department. Ultrasound diagnosis of teratomas has high sensitivity and specificity. Mucinous cystadenomas also have distinct sonographic features. In our case, an ultrasound and CT scan pelvis favored a diagnosis of mature cystic teratoma [9].

On gross appearance, most of these tumors are large, unilateral, and cystic. They are characterized by a smooth ovarian surface comprising multiple cystic spaces with variable diameters [5]. Our patient presented with a unilateral ovarian cystic mass of 11 x 10 cm with multiple locules with torsion. Mucinous epithelium without papillary infoldings is typical of mucinous cyst adenomas. According to WHO recommendations, the criteria to qualify as a borderline mutinous tumor include at least 10% of the epithelial volume, which should demonstrate increased proliferation with papillary infoldings or pseudo stratification, along with mild to moderate nuclear atypia.

By immunohistochemistry, these borderline tumors demonstrate diffuse expression of cytokeratin 7, patchy co-expression of cytokeratin 20, and variable (usually weak) expression of CDX2 in nearly 40% of patients. It is a fact that cytokeratin (CK) 7/CK 20 expression profiles are very useful for distinguishing primary ovarian mucinous tumors from metastases arising from the lower intestinal tract (appendix, colorectum), as a majority exhibit diffuse expression of CK 20 coupled with lacking/limited expression of CK 7. In contrast, primary mucinous tumors of the gastrointestinal tract involving the ovaries often exhibit diffuse expression of CK 7 coupled with variable expression of CK 20 that is often present but usually patchy and not diffuse [10].

In our case, immunohistochemically, CK20 expression was mucinous tumor epithelium positive, and CK7 expression was mucinous tumor negative. This result suggests that the tumor could have originated from the solid area of the ovarian lesion. Very few previous studies reported the co-existence of mature cystic teratoma with borderline mucinous cystadenoma [9]. Our patient also had a mature cystic teratoma with mucinous cystadenoma with focal intraepithelial carcinoma.

## Conclusions

Ovarian tumors can present in different ways. As a clinician, we should have a high index of clinical suspicion considering the age, size, presentation, radiological features of the ovarian tumor, and tumor marker reports to treat the ovarian teratoma with possible malignant transformation definitively. This case is reported as a rare occurrence of mature cystic teratoma presented as torsion, postoperatively diagnosed by histopathology and immunochemistry as a mature cystic teratoma with borderline mucinous cystadenoma and focal intra-epithelial malignant transformation. In our case, we could diagnose ovarian intraepithelial carcinoma at an early stage, which has a better prognosis. This conclusion is in line with the currently available literature and evidence.
